# Inspiratory Muscle Weakness is Related to Poor Short-Term Outcomes for Heart Transplantation

**DOI:** 10.21470/1678-9741-2020-0344

**Published:** 2021

**Authors:** Isis Begot, Walter J. Gomes, Isadora S. Rocco, Caroline Bublitz, Laion R. A. Gonzaga, Douglas W. Bolzan, Vinicius Batista Santos, Rita Simone Lopes Moreira, João R. Breda, Dirceu Rodrigues de Almeida, Ross Arena, Solange Guizilini

**Affiliations:** 1 Cardiology Postgraduate Program, Federal University of Sao Paulo, Sao Paulo, Sao Paulo, Brazil.; 2 Cardiology and Cardiovascular Surgery Disciplines, Sao Paulo Hospital, Escola Paulista de Medicina, Federal University of Sao Paulo, Sao Paulo, Sao Paulo, Brazil.; 3 Paulista School of Nursing, Federal University of Sao Paulo, Sao Paulo, Brazil.; 4 Department of Physical Therapy, College of Applied Health Sciences, University of Illinois at Chicago, Chicago, Illinois, United States of America.; 5 Department of Human Motion Sciences, Federal University of Sao Paulo, São Paulo, Brazil.

**Keywords:** Heart Failure, Heart Transplantation, Respiratory Muscle, Muscle Weakness, Treatment Outcomes

## Abstract

**Introduction:**

In heart transplantation (HT) recipients, several factors are critical to promptly adopting appropriate rehabilitation strategies and may be important to predict outcomes way after surgery. This study aimed to determine preoperative patient-related risk factors that could adversely affect the postoperative clinical course of patients undergoing HT.

**Methods:**

Twenty-one hospitalized patients with heart failure undergoing HT were evaluated according to respiratory muscle strength and functional capacity before HT. Mechanical ventilation (MV) time, reintubation rate, and intensive care unit (ICU) length of stay were recorded, and assessed postoperatively.

**Results:**

Inspiratory muscle strength as absolute and percentpredicted values were strongly correlated with MV time (r=-0.61 and r=-0.70, respectively, at P<0.001). Concerning ICU length of stay, only maximal inspiratory pressure (MIP) absolute and percent-predicted values were significantly associated. The absolute |MIP| was significantly negatively correlated with ICU length of stay (r=-0.58 at P=0.006) and the percent-predicted MIP was also significantly negatively correlated with ICU length of stay (r=-0.68 at P=0.0007). No associations were observed between preoperative functional capacity, age, sex, and clinical characteristics and MV time and ICU length of stay in the cohort included in this study. Patients with respiratory muscle weakness had a higher prevalence of prolonged MV, reintubation, and delayed ICU length of stay.

**Conclusion:**

An impairment of preoperative MIP was associated with poorer short-term outcomes following HT. As such, inspiratory muscle strength is an important clinical preoperative marker in patients undergoing HT.

**Table:** 

Abbreviations, acronyms & symbols	
6MWT	= Six-minute walk test
ATS	= American Thoracic Society
CPB	= Cardiopulmonary bypass
HF	= Heart failure
HT	= Heart transplantation
ICU	= Intensive care unit
LVEF	= Left ventricular ejection fraction
MIP	= Maximal inspiratory pressure
MV	= Mechanical ventilation
NYHA	= New York Heart Association
PASP	= Pulmonary arterial systolic pressure

## INTRODUCTION

Heart failure (HF) is a highly prevalent global syndrome, especially in older subjects, and represents a major cause of hospitalization, morbidity, and mortality^[1]^. Heart transplantation (HT) is considered the gold standard for the treatment of refractory terminal HF, especially since the 1980s, with the advent of immunosuppressive therapy^[2]^.

In HT recipients, accurate identification of all factors that may affect outcome in terms of functional recovery, morbidity, and mortality is critical to promptly adopt appropriate rehabilitation strategies^[3]^. Left ventricular dysfunction is related to systemic alterations and is an important marker to stratify risk for exercise-induced events during rehabilitation^[4]^. As such, advanced HF leads to alteration of pulmonary pressure due to chronic congestion secondary to left chamber dysfunction. Preoperative pulmonary hypertension has been described as a prognostic marker for short-term outcomes after HT^[5]^.

Patients with HF have an impaired functional capacity, *i.e*., poor six-minute walk test (6MWT) distance, poor quality of life, and muscle weakness; deconditioning plays an important role in these clinical characteristics. Interestingly, in addition to peripheral muscle impairment, the respiratory musculature is also oftentimes compromised in patients with HF^[6]^. Previous work has demonstrated a relationship between decreasing maximal inspiratory muscle strength and endurance and metabolic and structural damage to diaphragmatic fibers^[7]^.

Meyer et al.^[7,8]^has shown that respiratory muscle strength has been characterized as an independent predictor of HF prognosis. The reduction of respiratory muscle strength, as determined by maximal inspiratory pressure (MIP), is related to the severity of HF, as demonstrated by the stepwise worsening impairment in MIP with increasing New York Heart Association (NYHA) functional class^[9]^. In this context, we hypothesized that clinical baseline characteristics, decrease functional capacity, and respiratory muscle weakness may contribute to the development of poor postoperative outcomes of HF patients undergoing HT.

If our hypothesis is confirmed, these measures could guide more precise therapeutic and rehabilitative strategies in these patient populations^[3]^. As such, this study aimed to determine preoperative patient-related risk factors that could adversely affect the postoperative clinical course in patients undergoing HT.

## METHODS

This observational study was conducted between October 2013 and February 2017 at the Hospital São Paulo, Hospital Universitário da Universidade Federal de São Paulo, Brazil. All appropriate ethical aspects were followed with study approval obtained from the institution’s Clinical Ethical Research Committee. All subjects were informed about the study and signed a written consent form before enrolment.

### Subjects

For this prospective study, hospitalized patients with end-stage HF undergoing HT were recruited. Eligible criteria were applied as follows: 1) both sexes; 2) age between 18 and 70 years; and 3) an HF diagnosis determined by the referring clinician, confirmed by echocardiography and stratified according to clinical presentation (NYHA classes III and IV).

Exclusion criteria applied were: 1) chronic obstructive pulmonary disease, confirmed by spirometry according to the Global Initiative for Obstructive Lung Disease, or GOLD^[10]^; 2) unstable angina pectoris; 3) atrial and ventricular arrhythmias leading to hemodynamic compromise; 4) hemodynamic instability; 5) acute coronary syndromes; 6) chronic renal failure or dialysis; 7) intraoperative death; 8) neuromuscular and psychiatric conditions that could potentially influence test performance; and 9) noncardiac conditions limiting exercise performance.

### Surgical Procedures

During the intraoperative period, all patients were submitted to the same protocol of anesthesia and mechanical ventilation (MV). The surgical procedure followed a standard protocol, with median sternotomy access and usual cannulation for cardiopulmonary bypass (CPB). After the onset of CPB, recipient cardiectomy was performed so that it could be completed simultaneously with the arrival of the donor heart. All operations were performed using the bicaval technique^[11]^, first sequencing left atrial anastomosis, usually followed by anastomosis of the inferior vena cava, pulmonary artery, aorta, and superior vena cava. At the end of the surgery the usual mediastinal and pleural thoracic drains were placed. Before closing the sternum, atrial and ventricular temporary pacing wires were inserted.

After the procedure, all patients were transferred to the intensive care unit (ICU) where postoperative care was performed in a manner similar to that of other open-heart surgery cases. Extubation was performed according to an established protocol in the ICU, followed by noninvasive MV for one hour. The mediastinal tubes were removed as early as possible, according to the rate of fluid drainage. The immunosuppression protocol consisted of cyclosporine, azathioprine, and methylprednisolone. All patients underwent endomyocardial biopsy to monitor acute rejection during the in-hospital phase.

### Respiratory Muscle Strength

An analogical manometer (Critical Med, Rio de Janeiro, Rio de Janeiro, Brazil) was used for determination of preoperative MIP. This protocol was performed as described by the American Thoracic Society (ATS)^[12]^ and reference values were obtained through equations described by Neder et al.^[13]^.

### Functional Capacity

Functional capacity was determined by the 6MWT performed in the preoperative period according to ATS Guidelines^[14]^ and reference values were obtained through equations described by Soares-Pereira et al.^[15]^.

### Patient-Related Factors and Clinical Outcomes

Anthropometric data were obtained through the admission and clinical data by echocardiography (*i.e*., preoperative pulmonary arterial systolic pressure [PASP] and left ventricular ejection fraction [LVEF]). The MV time, reintubation rate, length of postoperative ICU stay, and mortality were recorded for all patients.

### Statistical Analysis

The normality distribution of data was analyzed by the Kolmogorov-Smirnov test. Categorical data were presented in absolute (n) and relative (%) frequency. Semi-continuous and continuous variables were presented as mean and standard deviation. The Pearson’s correlation coefficient was used to determine the association between age, inspiratory muscle strength, PASP, LVEF, and the 6MWT with MV time and length of postoperative ICU stay.

Frequency distributions for patients with and without inspiratory muscle weakness (MIP < 70% of predicted value) in respect of outcomes were obtained as follows: postoperative prolonged MV time (> 48 hours), reintubation, and prolonged ICU length of stay (> 6 days). A *P*-value < 0.05 was used to consider statistical significance for all tests.

## RESULTS

**Fig. 1:**
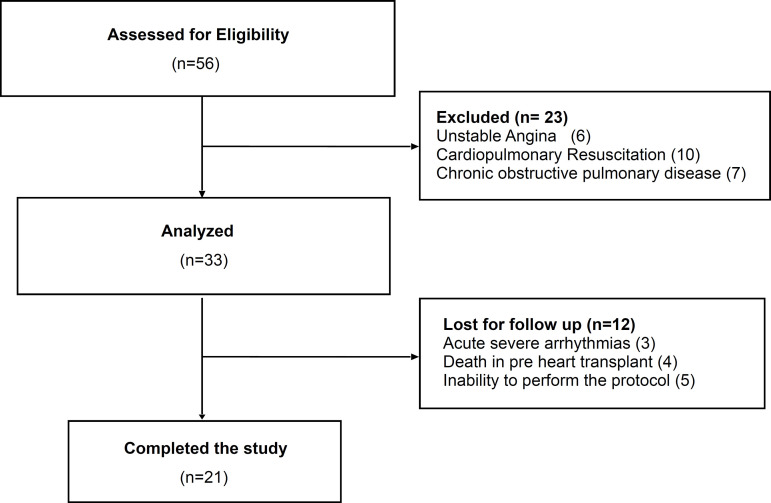
Flowchart of the study evaluation protocol.

During the study period, 56 patients were assessed for eligibility, and from that sample, 21 patients were included. The progression of patients throughout the study is indicated in a flowchart (Figure 1). Baseline clinical and anthropometric characteristics of participants are summarized in Table 1.

**Table 1:** Clinical and anthropometric characteristics.

Variables		n=21
Age (years)		50.09±10.8
Gender (n)	Male/Female	15/6
	LVEF (%)	26.09±7.70
PASP (mmHg)		47.33 (10.53)
Etiology of HF	Ischemic, % (n)	20 (4)
	Chagas disease, % (n)	40 (8)
	Valvar disease, % (n)	15 (3)
	Others, % (n)	28 (6)
	6MWT (meters)	255.93±80.69
	Percent-predicted	45.36±13.85
	|MIP| (cmH2O)	77.62±25.08
	Percent-predicted	73.43±21.73
	Inspiratory weakness, % (n)	42.9 (9)
	CPB time (minutes)	132.8±21.1
	MV time (hours)	110.9±11.5
	Length of postoperative ICU stay (days)	10.7±6.6
Drug therapy pre-HT	Angiotensin-converting enzyme inhibitors (mg/day)	20.2±16.74
	Furosemide (mg/day)	30.45±10.24
	Dobutamine (µg/kg/min)	8.02±3.81

Data expressed as mean ± standard deviation

6MWT=six-minute walk test; CPB=cardiopulmonary bypass; HF=heart failure; HT=heart transplantation; ICU=intensive care unit; LVEF=left ventricular ejection fraction; MIP=maximal inspiratory pressure; MV=mechanical ventilation; PASP=pulmonary arterial systolic pressure

**Fig. 2:**
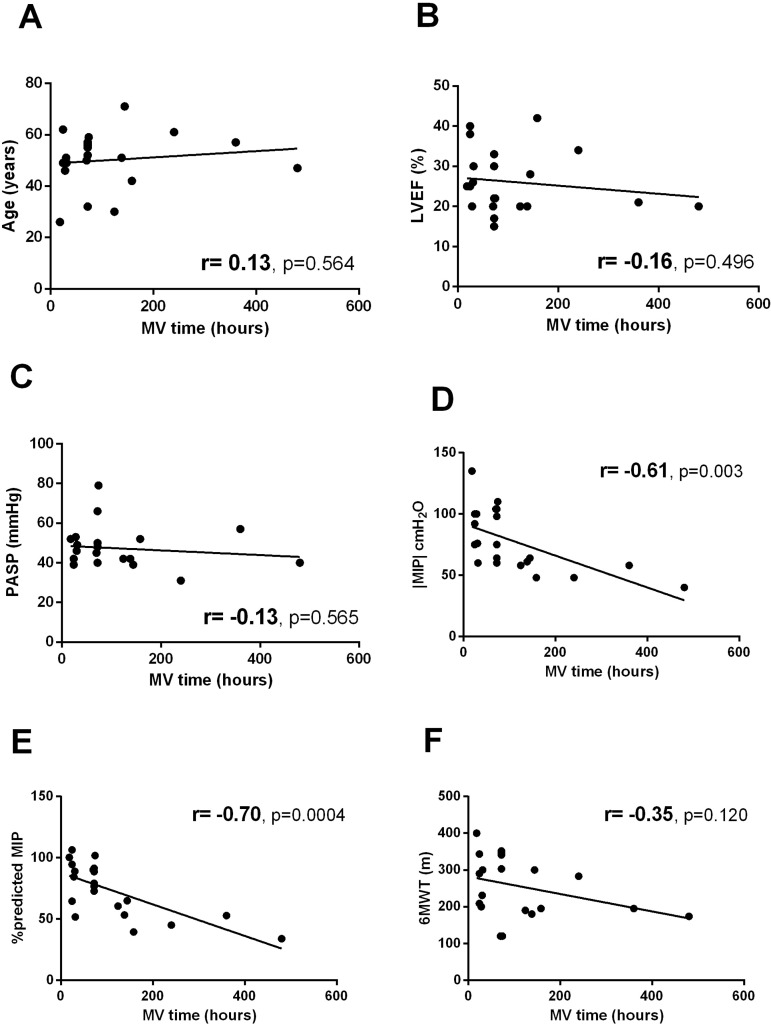
Clinical factors related to mechanical ventilation (MV) time. A. Age; B. pulmonary arterial systolic pressure (PASP); C. left ventricular ejection fraction (LVEF); D. maximal inspiratory pressure (MIP); E. percent-predicted MIP; F. six-minute walk test (6MWT) distance.

**Fig. 3:**
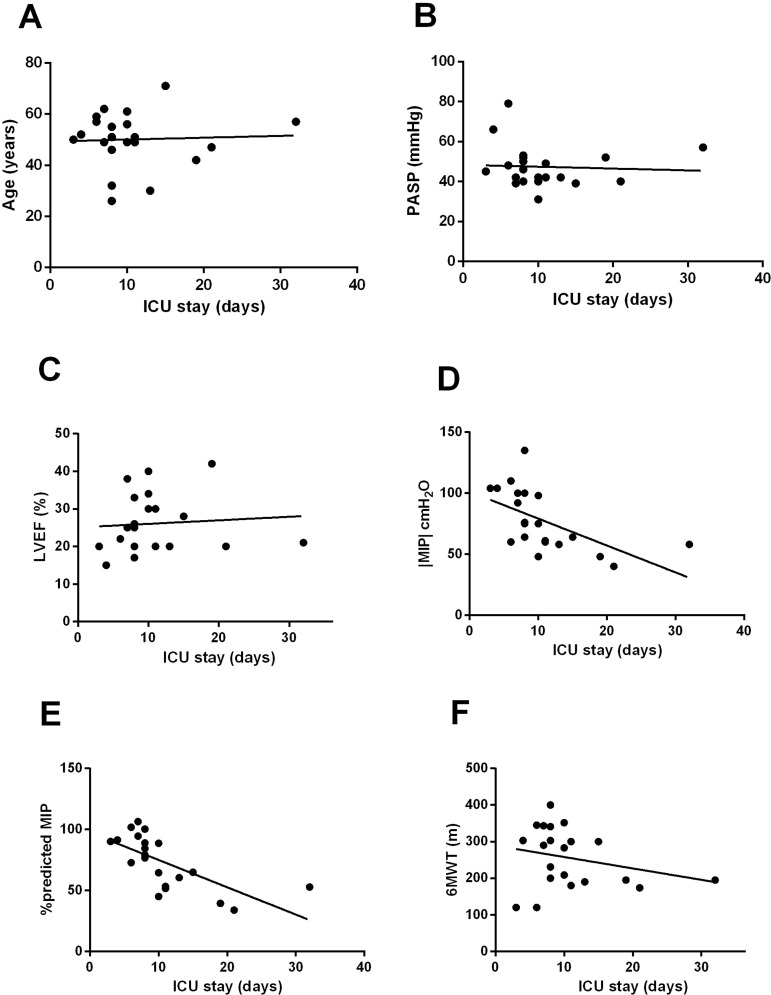
Clinical factors related to length of intensive care unit (ICU) stay. A. Age; B. pulmonary arterial systolic pressure (PASP); C. left ventricular ejection fraction (LVEF); D. maximal inspiratory pressure (MIP); E. percent-predicted MIP; F. six-minute walk test (6MWT) distance.

The inspiratory muscle strength as absolute and percent-predicted values were significantly negatively correlated with MV time (r=-0.61 with *P*=0.0031 and r=-0.70 with *P*=0.0004, respectively, Figure 2). Concerning ICU length of stay, only absolute and percent-predicted |MIP| values were significantly associated. Absolute |MIP| was significantly negatively correlated with ICU length of stay (r=-0.58 with *P*=0.006, Figure 3), and the percent-predicted MIP was also significantly negatively correlated with ICU length of stay (r=-0.68 with *P*=0.0007, Figure 3). No associations were observed between age, PASP, LVEF, or 6MWT and MV time and length of postoperative ICU stay.

When patients were dichotomized according to the presence of respiratory muscle weakness, those with percent-predict MIP < 70% presented a higher prevalence of prolonged MV, and significantly higher prevalence of reintubation, and delayed ICU discharge (Table 2).

**Table 2:** Patients with or without respiratory muscle weakness regardless of short-term outcomes.

Events	Inspiratory muscle weakness		*P*-value
	With (n=9)	Without (n=12)	
Prolonged MV, n (%)	7 (78)	4 (33)	0.04
Reintubation, n (%)	3 (33)	0 (0)	0.03
Prolonged length of ICU stay, n (%)	8 (89)	4 (33)	0.02
Death, n (%)	3 (33)	2 (17)	0.37

ICU=intensive care unit; MV=mechanical ventilation

Five deaths were observed within 30 days of HT. From this sample, three of those patients had preoperative inspiratory muscle weakness.

## DISCUSSION

The results of the current study revealed that the presence of inspiratory muscle weakness was associated with poorer clinical outcomes following HT. Preoperative MIP evaluation demonstrated clinical value that is easily performed, noninvasive, and safe to administer in the inpatient environment^[16]^.

Although MIP depends on patients’ cooperation and maximal effort, repeated measurements reveal good reproducibility^[16]^. This test could be easily applied in all inpatient clinical settings, administered at the same time as a routine pulmonary function test or as a standalone measure. Another advantage is the possibility to risk stratification of patients independently of their ability to walk or cycle.

Impaired respiratory muscle strength is frequently present in HF patients, possibly reflecting an increased work of breathing. Chronic lung congestion occurs secondarily to left ventricle dysfunction causing a restrictive ventilatory pattern and inefficiency, overloading the respiratory muscles in HF patients^[17]^.

Beyond mechanical mechanism, inspiratory muscle weakness can occur due to deconditioning status, indicating the role of skeletal muscle proteolysis. A generalized skeletal muscle disorder is associated with oxidative stress and activation of pro-inflammatory pathways frequently present in HF patients^[18,19]^. This process is described as sarcopenia, a systemic skeletal muscle disorder that impairs the function of both the skeletal and respiratory musculature, resulting in further functional decline^[20]^. Also, a switch of muscle fiber types is observed as HF severity increases^[21]^. Tukinov et al.^[18]^ investigated skeletal muscle myopathy in HF patients, finding a greater percentage of type I muscle fibers and a significantly lower percentage of type II and type IIa muscle fibers in the costal diaphragm compared to healthy subjects. A disease-induced adaptation of type I fiber predominance revealed poorer respiratory strength and power secondary to the pathophysiologic effects of HF^[19]^. The authors suggested that adding assessments for inspiratory muscle power could more completely detect the impact of HF on muscle dysfunction. In fact, our results revealed that 42.9% of patients with terminal HF had respiratory muscle weakness, defined as an MIP < 70% of predicted. This finding is corroborated by previous studies that indicate a 30-50% prevalence of respiratory muscle weakness in HF patients^[22]^.

A longer time of exposure to HF in conjunction with aging could precipitate further complications. Studies have shown a progressive reduction of |MIP| between 0.8 and 2.7 cmH_2_O per year in elderly subjects^[23,24]^. Elderly subjects also often present with cognitive impairment, frailty, and a significant number of other comorbidities. Therefore, age is a common risk factor for cardiovascular events and mortality, in clinically and surgically managed patients with HF. However, the findings of our study did not show an association between age and MV time or ICU length of stay. These results may indicate that, in patients with HF undergoing HT, other age-independent mechanisms could better account for short-term risk following HT.

A reduction in LVEF explains cardiopulmonary mechanical disarrangement and systemic alterations in HF patients. Previous studies demonstrated that inpatients with reduced LVEF exhibit poor functional capacity and worsening respiratory muscle strength^[25]^. Nevertheless, HF patients develop compensatory systemic mechanisms, such as tolerance to high pulmonary vascular pressure during effort and peripheral vascular resistance control, which are not necessarily related to the degree of LVEF^[26]^. Although LVEF had been recognized as an important prognostic marker following heart surgery^[27]^, the current findings indicate LVEF was not related to other measures of interest. Even though left ventricular dysfunction may display systemic alterations, the compensatory mechanisms are more related to prognosis than LVEF itself, especially in HT, where the failing heart is replaced. Similarly, in the present study, our results showed that LVEF was not related to MV time and ICU length of stay. Meyer et al.^[8]^ demonstrated that the prognostic value of LVEF could be improved by adding the assessment of MIP and peak of oxygen uptake in HF patients. They also found that MIP was able to predict prognosis independently of NYHA class and norepinephrine levels.

To meet the unique mechanisms underlying the course of HF, recent studies have addressed the importance of pulmonary hypertension in HF. Bursi et al.^28]^ (2012) demonstrated that PASP strongly predicts mortality and is an incremental and clinically relevant prognostic marker independent of other established predictors of outcome in HF patients. Within the possibility of vascular modulation in the presence of pulmonary hypertension, the present study investigates whether PASP would be associated with short-term outcomes following HT. However, in the present study, we did not observe a correlation between these variables, which could be explained by the advanced HF profile in our cohort patients. Specifically, the patients included in the current study had severe HF necessitating HT, and therefore higher levels of PASP.

The severity of disease progression in the current study can be confirmed by the patients with the NYHA functional class around III and IV. Filusch et al.^[29]^ (2011) demonstrated, in a large cohort of HF patients (n=5,532), that inspiratory muscle strength was progressively more impaired according to the worsening NYHA functional class. They identified that patients with NYHA class I have significantly greater strength compared to those of all other NYHA classes. Whereas patients with NYHA class IV have significantly poorer respiratory muscle power compared to those in all other NYHA classes^[9,19]^.

These assumptions reveal that patients with worse NYHA and lower respiratory muscle strength had impairment in functional capacity and exercise performance. A well-established and accepted evaluation of functional capacity in HF patients undergoing HT is the 6MWT. Frequently employed in clinical practice, the 6MWT is an easy-to-perform test, has the ability to differentiate the clinical impact of therapies, holds prognostic value, and is better tolerated than a maximal incremental exercise test^[30]^.

Stewart et al.^[31]^ (2014) indicated that patients with left ventricular dysfunction who walked < 300 meters in the preoperative period were at higher risk of unfavorable outcomes following myocardial revascularization surgery. Notwithstanding, the findings of the current study did not observe an association between 6MWT distance and MV time or ICU length of stay. Likewise, Rocco et al.^[32]^ (2018) identified that the 6MWT alone is not able to predict short-term outcomes following myocardial revascularization, unless patients had an obvious impairment in 6MWT distance. They found that direct measurement of oxygen consumption evaluation would more accurately predict risk during the postoperative period, especially because ventilatory expired gas analysis provides more substantial data about compensatory mechanisms, tolerance of high arterial pulmonary pressures, and ventilatory performance.

Previous studies confirmed the association between ventilatory inefficiency, measured by the ratio between ventilation and carbon dioxide production, and inspiratory muscle weakness, verified by low MIP^[33]^. Several protocols of inspiratory muscle training were able to improve ventilatory efficiency, especially in those patients with advanced HF^[20,34]^.

Regardless of the complexity of parameters evaluated in the preoperative period of HT, inspiratory muscle strength (*i.e*., MIP) seems to reflect an overall adaptation secondary to refractory HF. The current study identified that the main parameter able to detect higher chances of short-term outcomes following HT is MIP. Patients with respiratory muscle weakness, *i.e*., percent-predicted MIP < 70%, expressed a higher prevalence of reintubation, prolonged MV, and delayed ICU length of stay.

In the postoperative phase of cardiac surgery, prolonged MV time is currently defined as a time longer than 24 to 48 hours of MV support^[35]^. In HF patients within the preoperative phase of HT, marked respiratory muscle weakness is already established before MV^[25]^. A previous report identified that respiratory muscle training in the preoperative phase benefits patients in the postoperative phase of revascularization surgery, with a lower incidence of pulmonary complications, including MV dependence^[36]^. These assumptions suggest that a higher impartment of MIP in the preoperative phase is associated with prolonged MV time postoperatively.

To the best of our knowledge, our study was the first to directly investigate the association between preoperative respiratory muscle weakness and postoperative short-term outcomes in hospitalized patients undergoing HT. Camkiran et al.^[37]^ (2015) analyzed patients in the postoperative phase of HT and found a longer MV time (123.8 hours), and, consequently, a prolonged ICU stay (19.8 days). This study corroborates with our findings, where our patients with a longer MV time (110.9 hours) also evolved with a prolonged ICU stay (10.7 days).

A previous study found a negatively significant association between MIP and C-reactive protein, fibrinogen, and white blood cell count. Beyond mechanical compensation, the investigators of the same study concluded that inspiratory muscle weakness may be a marker of metabolic and inflammatory pathophysiologic processes^[19,38]^.

The current scenario expresses the importance of inspiratory muscle weakness as a clinical manifestation of the severity of HF adaptations, with an interdependence between cardiac and respiratory systems, and also indicates that it is an important prognostic tool^[18,19]^. Evidence suggests that respiratory muscle strength improvement before coronary artery bypass graft surgery was able to reduce the incidence of postoperative pulmonary complications, MV time, and length of hospital stay^[39]^. The present findings support that increasing inspiratory muscle strength in the preoperative phase may also improve early postoperative outcomes in patients undergoing HT.

### Limitations

A limitation of the present study was the absence of a greater number of patients. As such, we had a limited number of deaths and were unable to perform an in-depth and conclusive survival analysis.

## CONCLUSION

Impairment in preoperative MIP was associated with poorer short-term outcomes following HT. Therefore, our findings in conjunction with previous research indicate that inspiratory muscle strength appears to be an important clinical measure in patients undergoing HT.

**Table:** 

Authors' roles & responsibilities	
IB	Substantial contributions to the conception or design of the work; or the acquisition, analysis, or interpretation of data for the work; drafting the work or revising it critically for important intellectual content; agreement to be accountable for all aspects of the work in ensuring that questions related to the accuracy or integrity of any part of the work are appropriately investigated and resolved; final approval of the version to be published
WJG	Substantial contributions to the conception or design of the work; or the acquisition, analysis, or interpretation of data for the work; drafting the work or revising it critically for important intellectual content; agreement to be accountable for all aspects of the work in ensuring that questions related to the accuracy or integrity of any part of the work are appropriately investigated and resolved; final approval of the version to be published
ISR	Substantial contributions to the conception or design of the work; or the acquisition, analysis, or interpretation of data for the work; drafting the work or revising it critically for important intellectual content; agreement to be accountable for all aspects of the work in ensuring that questions related to the accuracy or integrity of any part of the work are appropriately investigated and resolved; final approval of the version to be published
CB	Substantial contributions to the conception or design of the work; or the acquisition, analysis, or interpretation of data for the work; drafting the work or revising it critically for important intellectual content; agreement to be accountable for all aspects of the work in ensuring that questions related to the accuracy or integrity of any part of the work are appropriately investigated and resolved; final approval of the version to be published
LRAG	Substantial contributions to the conception or design of the work; or the acquisition, analysis, or interpretation of data for the work; drafting the work or revising it critically for important intellectual content; agreement to be accountable for all aspects of the work in ensuring that questions related to the accuracy or integrity of any part of the work are appropriately investigated and resolved; final approval of the version to be published
DWB	Substantial contributions to the conception or design of the work; or the acquisition, analysis, or interpretation of data for the work; drafting the work or revising it critically for important intellectual content; agreement to be accountable for all aspects of the work in ensuring that questions related to the accuracy or integrity of any part of the work are appropriately investigated and resolved; final approval of the version to be published
VBS	Substantial contributions to the conception or design of the work; or the acquisition, analysis, or interpretation of data for the work; drafting the work or revising it critically for important intellectual content; agreement to be accountable for all aspects of the work in ensuring that questions related to the accuracy or integrity of any part of the work are appropriately investigated and resolved; final approval of the version to be published
RSLM	Substantial contributions to the conception or design of the work; or the acquisition, analysis, or interpretation of data for the work; drafting the work or revising it critically for important intellectual content; agreement to be accountable for all aspects of the work in ensuring that questions related to the accuracy or integrity of any part of the work are appropriately investigated and resolved; final approval of the version to be published
JRB	Substantial contributions to the conception or design of the work; or the acquisition, analysis, or interpretation of data for the work; drafting the work or revising it critically for important intellectual content; agreement to be accountable for all aspects of the work in ensuring that questions related to the accuracy or integrity of any part of the work are appropriately investigated and resolved; final approval of the version to be published
DRA	Substantial contributions to the conception or design of the work; or the acquisition, analysis, or interpretation of data for the work; drafting the work or revising it critically for important intellectual content; agreement to be accountable for all aspects of the work in ensuring that questions related to the accuracy or integrity of any part of the work are appropriately investigated and resolved; final approval of the version to be published
RA	Substantial contributions to the conception or design of the work; or the acquisition, analysis, or interpretation of data for the work; drafting the work or revising it critically for important intellectual content; agreement to be accountable for all aspects of the work in ensuring that questions related to the accuracy or integrity of any part of the work are appropriately investigated and resolved; final approval of the version to be published
SG	Substantial contributions to the conception or design of the work; or the acquisition, analysis, or interpretation of data for the work; drafting the work or revising it critically for important intellectual content; agreement to be accountable for all aspects of the work in ensuring that questions related to the accuracy or integrity of any part of the work are appropriately investigated and resolved; final approval of the version to be published
